# The impact of early emergency department allied health intervention on admission rates in older people: a non-randomized clinical study

**DOI:** 10.1186/1471-2318-12-8

**Published:** 2012-03-20

**Authors:** Glenn Arendts, Sarah Fitzhardinge, Karren Pronk, Mark Donaldson, Marani Hutton, Yusuf Nagree

**Affiliations:** 1 Centre for Clinical Research in Emergency Medicine, Western Australian Institute for Medical Research, Level 5 MRF Building, Rear 50 Murray St, WA 6000 Perth, Australia; 2 School of Primary, Aboriginal and Rural Health Care, University of Western Australia, Perth, Australia; 3 Department of Emergency Medicine, Royal Perth Hospital, Perth, Australia; 4 Department of Geriatric Medicine, Royal Perth Hospital, Perth, Australia; 5 South Metropolitan Area Health Service, Western Australian Department of Health, Perth, Australia; 6 Department of Emergency Medicine, Fremantle Hospital, Fremantle, Australia

## Abstract

**Background:**

This study sought to determine whether early allied health intervention by a dedicated Emergency Department (ED) based team, occurring before or in parallel with medical assessment, reduces hospital admission rates amongst older patients presenting with one of ten index problems.

**Methods:**

A prospective non-randomized trial in patients aged sixty five and over, conducted in two Australian hospital EDs. Intervention group patients, receiving early comprehensive allied health input, were compared to patients that received no allied health assessment. Propensity score matching was used to compare the two groups due to the non-randomized nature of the study. The primary outcome was admission to an inpatient hospital bed from the ED.

**Results:**

Of five thousand two hundred and sixty five patients in the trial, 3165 were in the intervention group. The admission rate in the intervention group was 72.0% compared to 74.4% in the control group. Using propensity score probabilities of being assigned to either group in a conditional logistic regression model, this difference was of borderline statistical significance (*p *= 0.046, OR 0.88 (0.76-1.00)). On subgroup analysis the admission rate in patients with musculoskeletal symptoms and angina pectoris was less for those who received allied health intervention versus those who did not. This difference was significant.

**Conclusions:**

Early allied health intervention in the ED has a significant but modest impact on admission rates in older patients. The effect appears to be limited to a small number of common presenting problems.

## Background

Presentations to the emergency department (ED) are increasing at a rate that exceeds population growth [1]. This increase is across all age ranges but highest in older people (defined here as aged 65 and over) [2]. As age increases the likelihood of admission into the hospital when a person presents to the ED also increases, with hospitalisation rates in older people two to four times higher than rates in younger adults [3,4].

Whilst most hospital admissions in older people from the ED are clinically appropriate, a minority are considered avoidable for want of multidisciplinary physician, nursing and allied health care that facilitates safe discharge and management in the community [5]. The clinical risks associated with hospitalisation of older people are well documented including deconditioning, functional decline, delirium and iatrogenic illness [6,7]. There are also potential benefits in terms of cost savings and improved patient satisfaction through avoiding hospital admission [8]. Several prior non-experimental studies have reported favourable results from the use of geriatric services, including allied health staff, to facilitate discharge from the ED [9-11]. Patients are predominantly referred to these services after ED assessment has deemed the patient potentially suitable for discharge, in other words the services are seen as enablers to assist discharge in patients deemed medically suitable for such.

The objective of this study was to assess whether early multidisciplinary allied health targeting of specific diagnoses, undertaken before ED medical assessment had been completed or even commenced, would change admission rates in older patients presenting to the ED with these problems. We hypothesised that the front loading of specialised geriatric allied health services provided by a care coordination team (CCT) would increase the likelihood of ED discharge (and so reduce admission rates) in people with these selected conditions.

## Methods

A non-randomized prospective study comparing patients that underwent CCT intervention to contemporaneous controls. The study was conducted from February 2009 to March 2010 in two tertiary hospital EDs with similar staffing profiles and internal configurations, one an exclusively adult ED with an annual census of approximately 65000, the other a mixed adultpaediatric ED with an annual census around 45000. Institutional ethics committee approval was obtained that included a provision for informed patient consent for the intervention (South Metropolitan Area Health Service HREC Reference 08/318 and Royal Perth Hospital HREC Reference 08/009).

Patients aged 65 and over presenting to the ED with one or more from a list of ten presenting medical complaints were included (Table 1). These diagnoses were selected on the basis of a prior audit of hospital statistics showing they were common ED presenting problems with a high rate of admission and substantial use of allied health resources as an inpatient.

**Table 1:** Selected index conditions for trial

Infectious	Musculoskeletal	Cardiovascular	Neurological
Urinary infection	Fall with minor injury	Cardiac failure	Transient ischaemic attack

Respiratory tract infection	Hip or knee pain (no clinical fracture)	Angina pectoris	New onset confusion or delirium

	Back pain	Syncope	

**Figure 1:**
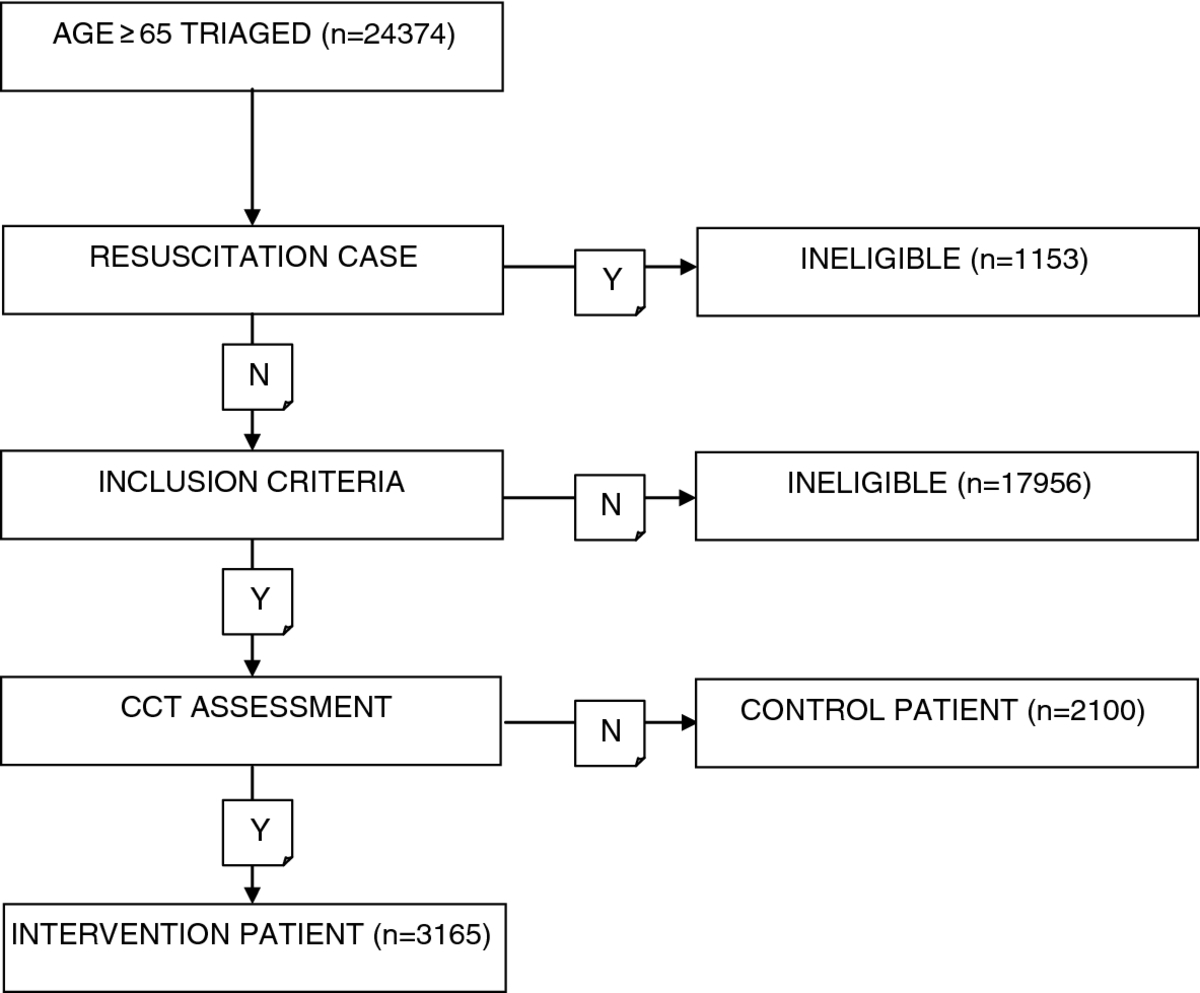
**Enrolment process**.

Eligibility and enrolment procedures for the study are summarised in Figure 1. Patients were screened early after triage on the basis of age and clinical presentation. Patients requiring immediate resuscitation, triage to a critical care bay in the ED or other urgent medical input were excluded.

The CCT were designed as exclusively ED based teams, functioning autonomously with a brief to facilitate safe early discharge to home of older people. In particular, they focussed on overcoming barriers to safe discharge whether they are related to the presenting problem (e.g. mobility post fall) or not (e.g. isolated unsupervised living arrangements in a patient with low grade cardiac failure). The CCT operated similarly at each site, teams were encouraged to select patients for assessment and intervention rather than wait for referral from other ED staff. Thus, team assessment occurred in parallel to ED medical staff assessment so that team input into disposition decisions for the patient could be incorporated into the final medical decision, always made by the treating ED physician, as to whether patient admission to hospital from ED was required. Each CCT contained at least one physiotherapist, occupational therapist and social worker with extensive geriatric experience. Physician (usually a geriatrician or geriatric trainee), nursing and other allied health staff such as speech therapists were co-opted to assist the teams as required. CCT were present in the ED seven days per week for a minimum of 10 h per day, but never between the hours of 2200 and 0600.

Intervention patients underwent comprehensive functional assessment by at least one CCT member, followed by the initiation of services to address any needs identified on assessment. The assessment incorporated components of previously validated instruments, modified after CCT input, for assessing factors such as discharge needs, falls risk, activities of daily living and cognition [12-14]. Control patients were eligible for CCT assessment but did not receive such before a decision to admit or discharge the patient was made.

Research assistants employed specifically for the study prospectively collected clinical data on intervention and control patients. Patient demographics, time of arrival and triage urgency were obtained from the Emergency Department Information System (EDIS)^®^. Admission to hospital was defined by an electronic bed request for admission to an inpatient ward bed including the ED observation ward. Deaths in the ED were included as admissions. Data were entered and stored electronically in SPSS^® ^version 17 (Chicago, IL, USA).

The study outcome was proportion of admissions to hospital from the ED. The intervention and control groups were compared on a univariate basis using Pearson's chi square test. Due to the non-randomized nature of the study, we used propensity score matching to stratify the likelihood of having been seen by CCT based on the presenting medical problem as well as potential confounders of gender, age, living arrangements, triage urgency, time of arrival to the ED, study site and comorbidities. Propensity scores provide a means for adjusting for bias in non-randomised studies of causal effects [15]. Effect modification (statistical interaction) was tested for and not found amongst the variables. The probability, based on their propensity score, of a patient being assigned to the intervention group was used in a nearest neighbour method to match intervention and control patients. Admission rates in the intervention and control groups were then compared using conditional logistic regression. A p value of 0.05 was used as the threshold for statistical significance. Pre-planned subgroup analysis on the basis of individual diagnoses, and logical groupings of diagnoses, was performed using binary logistic regression models adjusting for the potential confounders listed.

We had estimated that in the study period we would enrol 4000 patients evenly distributed between intervention and controls, which would provide 90% power to detect approximately a 5% difference in admission rates if the baseline rate was 66%.

## Results

In the study period there were 24374 presentations made by people aged 65 and over to the study sites, representing 19.8% of our total ED population in that time. Of these 1153 were resuscitation or critical cases; 17956 had presenting complaints that could not be related to the index diagnoses or had their entire ED stay occur outside of CCT working hours; leaving 5265 patients (3165 intervention and 2100 control) meeting inclusion criteria. Table 2 summarizes the study population. Table 3 shows the unadjusted comparison in admission between the intervention and control groups. There was a 2.4% absolute reduction in admissions in the intervention group (72% vs. 74.4%). Propensity score matching resulted in 61 groups with an average group size of 120 patients. When the probability, based on the propensity score, of a patient being assigned to the intervention or control group was included in a conditional logistic regression model the likelihood of admission in the intervention group was lower than the control group, with the difference of borderline statistical significance (OR 0.88, 95% CI 0.76-1.00, p 0.046).

**Table 2:** Summary of study population (3165 intervention and 2100 control patients)

Parameter		Intervention (n,% unless indicated)	Control (n,% unless indicated)
**Age (mean, SD)**		80,8	79,8

**Female**		1759,56	1135,54

**Living arrangements**	Independently alone	991,31	592,28

	Independently with other/s	1586,50	1157,55

	Assisted living facility	588,19	351,17

**Australasian Triage Scale**	2 (urgent)	1271,40	930,44

	3 (semi-urgent)	1198,38	839,40

	4/5 (not urgent)	696,22	331,16

**Charlson comorbidity index**	0	614,19	306,15

	1	684,22	445,21

	2+	1867,59	1349,64

**Table 3:** Unadjusted admission percentage in intervention versus control

	Intervention	Control	Total
**Admitted**	2279 (72.0%)	1563 (74.4%)	3842 (73.0%)

**Discharged**	886	537	1423

**Total**	3165	2100	5265

Tables 4 and 5 contain the adjusted OR for each individual condition and conditions combined into logical groupings. A beneficial CCT effect is seen with angina and musculoskeletal conditions, but no other diagnoses.

**Table 4:** Adjusted OR for admission in intervention group by individual targeted diagnoses

Diagnosis	N	Percent admitted	Adjusted OR (95% CI) for admission in intervention group	p
**Urinary infection**	407	59%	1.06 (0.68-1.64)	0.80

**Respiratory infection**	625	90%	1.09 (0.63-1.89)	0.75

**Fall minor injury**	320	55%	1.01 (0.56-1.82)	0.98

**Hip/knee pain**	428	73%	0.63 (0.37-1.06)	0.08

**Back pain**	229	53%	0.56 (0.29-1.08)	0.08

**Cardiac failure**	635	90%	1.19 (0.69-2.05)	0.54

**Angina**	1382	67%	0.71 (0.53-0.93)	0.01

**Syncope**	563	62%	0.98 (0.66-1.48)	0.94

**Transient ischaemic attack**	412	86%	0.65 (0.34-1.25)	0.20

**New delirium/confusion**	264	88%	1.66 (0.75-3.71)	0.21

**Table 5:** Adjusted OR for admission in intervention group by diagnostic group

Diagnostic Group	N	Percent admitted	Adjusted OR (95% CI) for admission in intervention group	p
**Infectious**	1032	77%	0.98 (0.71-1.35)	0.89

**Musculoskeletal**	977	62%	0.67 (0.49-0.93)	0.01

**Cardiovascular**	2580	72%	0.91 (0.75-1.10)	0.33

**Neurological**	676	87%	0.94 (0.58-1.54)	0.81

## Discussion

In patients aged 65 and over presenting to an ED, we have found that the early use of multidisciplinary allied health teams influences hospital admission rates in a small number of index diagnoses (angina and grouped musculoskeletal conditions) and these results were of borderline statistical significance. Our study, with over 5000 enrolments, is one of the largest trials of allied health practices in older people and adequately powered to detect the overall small difference found.

A number of studies have evaluated programs to look at ED discharge, largely from the perspective of reducing post-discharge risk. We are not aware of any study that has looked specifically at the impact of allied health staff on the admission decision. Enhanced identification of an at- risk discharged group and referral of that group to community care givers had some benefit in reducing short term functional decline post discharge [16]. More comprehensive geriatric assessment by additional care providers outside the primary care physician have shown conflicting results but have also found benefit on functional status [17], but not necessarily ED usage [18].

It appears that the benefit of CCT intervention on admission reduction is confined to musculoskeletal conditions such as back pain (OR 0.56) and lower limb pain (OR 0.63); or to conditions likely to be episodic and resolved by the time the patient has arrived in ED such as angina (OR 0.71). Our study shows no benefit accrues from CCT intervention in medical conditions with ongoing significant symptoms such as pneumonia, cardiac failure and delirium. Though these findings are robust, any subgroup analysis should be interpreted with caution due to the possibility of type II error. We had hypothesised that CCT input would reduce admission rates across all of the selected conditions studied. The lack of any intervention effect in conditions such as pneumonia or cardiac failure shows that almost all patients with these conditions presenting to ED are admitted, and that discharge of such patients is not enhanced by CCT input. The underlying medical condition of the patient rather than unmet community or functional needs is presumably more influential in the decision to admit in these subgroups. This has implications for workforce deployment; CCT like services should concentrate resources on patient groups where they are likely to produce a beneficial reduction in admission rather than those where admission rates are unchanged by their input.

We have confirmed that admission rates in the older ED patient are high. With population ageing a worldwide phenomenon, measures to safely reduce hospital admission rates in patients presenting to an ED are important in the quest to maximise efficient use of scarce inpatient beds. Even small changes in admission rates at the front door will result in meaningful reductions in hospital occupancy and improve system capacity. The use of allied health personnel working as a CCT in ED appears to support this aim.

In our study we included ED observation unit patients as admitted patients. Such patients typically have brief admissions of less than 24 h though there is evidence that at least 15% of such patients need longer inpatient stays [19]. By including short stay observation unit stays in the same group as inpatient admissions, we may have missed an additional benefit of CCT intervention if CCT increased the proportion of admissions that are observation unit admissions as opposed to ward admissions, where there is typically a longer length of stay. This was not part of our original hypothesis and so not analysed, but would be worthy of future research.

We have concentrated on admission avoidance, underpinned by an assumption of the clinical, economic and qualitative benefits of avoiding hospitalisation in carefully selected older patients. However, there are also risks involved with the discharge of older people from the ED - early functional decline, re-presentation and unanticipated death are all described [20-22]. In fact, other researchers have similarly used geriatric teams in ED to prevent inappropriate ED *discharge *[23]. The nature of a community's support services, the capacity of these services to consistently respond to referrals and the hospital's appetite for risk at point of discharge will all influence whether a patient is sent home from the ED. The decision to discharge or admit an older person, therefore, is sometimes a fine balance between the feasibility, risks and benefits of discharge.

Our study is noteworthy because we used assessment by CCT soon after arrival in the ED, before a decision on the disposition of the patient had been made, to assess the impact of early intervention on discharge rates. We did not attempt to assess the outcome of patients following discharge. Post discharge outcomes have been subject to a number of studies summarised in a recent systematic review which found some evidence of improved short term functional outcomes and reduced readmission rates with geriatric intervention at or after discharge [24]. A feature of these trials is that the referral to the geriatric team occurs after the decision to discharge has already been made [17].

In addition to admission avoidance, a number of other clinical benefits are likely to be derived from the location of experienced allied health teams within the ED. Studies have shown the functional needs of older people are often poorly recognised within the ED [25,26]. Communication between the ED and community care providers can be poor [27]. Coordination of care for patients with complex needs post discharge may be enhanced by specific planning [11].

Our study has several clear limitations. As the mechanism of allocation to intervention and control was not randomised, selection bias may have occurred. We attempted to adjust for this through the use of propensity score matching but this may have been insufficient. Because we have not included follow up data on patients that were discharged in our study, we cannot assume that patients discharged in the intervention group did not suffer a higher rate of short term adverse outcome compared to control group patients. However, the competence of allied health teams in facilitating safe discharge of ED patients has been well established in a systematic review of the literature [24], hence we sought to answer another question that had not previously been addressed. Because many factors influence decisions to discharge patients home from ED, the generalisability of our results to different settings or populations cannot be assumed. We have not included a formal cost effectiveness analysis in this paper but that is worthy of further research.

## Conclusions

Early allied health intervention in the ED has a borderline statistically significant but very modest impact on admission rates in older patients. The effect appears to be restricted to a small number of presenting problems that are episodic or musculoskeletal in nature.

## Competing interests

The authors declare that they have no competing interests.

## Authors' contributions

GA and MH conceived the idea for the study. GA designed the trial. GA, MH and YN supervised the conduct of the trial. SF and KP undertook recruitment and data collection and managed trial processes. GA wrote the manuscript and all other authors edited it. All authors participated in obtaining research funding and interpreting results. All authors read and approved the final manuscript.

## Pre-publication history

The pre-publication history for this paper can be accessed here:

http://www.biomedcentral.com/1471-2318/12/8/prepub
